# Polyhexamethylene Guanidine Phosphate Enhanced Procoagulant Activity through Oxidative-Stress-Mediated Phosphatidylserine Exposure in Platelets

**DOI:** 10.3390/toxics12010050

**Published:** 2024-01-08

**Authors:** Ju Hee Choi, Keunyoung Kim

**Affiliations:** College of Pharmacy, Kangwon National University, Chuncheon 24341, Republic of Korea; julie1345@naver.com

**Keywords:** polyhexamethylene guanidine phosphate (PHMG-p), platelets, phosphatidylserine (PS) exposure, procoagulant activation, humidifier disinfectant, cardiovascular disease

## Abstract

Polyhexamethylene guanidine phosphate (PHMG-p) is a common biocidal disinfectant that is widely used in industry and household products. However, PHMG-p was misused as a humidifier disinfectant (HD) in South Korea, which had fatal health effects. Various health problems including cardiovascular diseases were observed in HD-exposed groups. However, the potential underlying mechanism of HD-associated cardiovascular diseases is poorly understood. Here, we examined the procoagulant activity of platelets caused by PHMG-p and clarified the underlying mechanism. PHMG-p enhanced phosphatidylserine (PS) exposure through alteration of phospholipid transporters, scramblase, and flippase. Intracellular calcium elevation, intracellular ATP depletion, and caspase-3 activation appeared to underlie phospholipid transporter dysregulation caused by PHMG-p, which was mediated by oxidative stress and mitochondrial dysfunction. Notably, antioxidant enzyme catalase and calcium chelator EGTA reversed PHMG-p-induced PS exposure and thrombin generation, confirming the contributive role of oxidative stress and intracellular calcium in the procoagulant effects of PHMG-p. These series of events led to procoagulant activation of platelets, which was revealed as enhanced thrombin generation. Collectively, PHMG-p triggered procoagulant activation of platelets, which may promote prothrombotic risks and cardiovascular diseases. These findings improve our understanding of HD-associated cardiovascular diseases.

## 1. Introduction

Polyhexamethylene guanidine phosphate (PHMG-p), a polymeric guanidine family compound, has anti-bacterial and anti-fungal activity and is known to be less toxic than other common biocidal disinfectants [1,2,3,4,5]. The antimicrobial effects of PHMG are mediated by the disruption of the cell membrane through the cationic guanidine group interacting with negatively charged phosphatidylglycerol, an abundant membrane phospholipid in bacteria [6,7,8]. Due to its strong antimicrobial effects and safety profile, PHMG is widely used in industry, such as in agriculture as an antiseptic, animal husbandry, textiles, lumber sectors, medicinal sterilization, and water sanitation [3,9,10]. Moreover, PHMG has been extensively used in various household products including detergents, fabric softeners, paints, plastics, air conditioners, heaters, and humidifiers.

PHMG was misused as a major component of humidifier disinfectants (HDs), which had tragic effects in South Korea [11,12,13]. Epidemiological studies have shown its close association with lung injury, lung fibrosis, and pulmonary diseases in HD-exposed groups [14,15,16]. Therefore, PHMG is prohibited in spray-type products such as humidifier disinfectant, air freshener, and cleaning spray. However, PHMG is still considered for application in various other areas, and the potential risk should not be ignored.

Several studies raised the possibility of injuries in non-respiratory organs including the cardiovascular system, liver, brain, immune system, and skin [12,17]. In a study with 6061 HD victims, the prevalence of cardiovascular and cerebrovascular diseases increased 2.1- and 2.5-fold after HD exposure [13]. From the survey data of 4179 claimants for compensation from the Korea government, 203 (4.9%) claimants suffered from cardiovascular diseases including hypertension, heart failure, myocardial infarction, angina pectoris, peripheral vascular diseases, thrombosis, and atherosclerosis [18]. Moreover, in a comprehensive study with 1341 deceased victims, circulatory system diseases (84, 6.3%) were one of the most common causes of death after respiratory diseases and neoplasms [11]. However, few studies have focused on the effects in the cardiovascular system caused by major components of HD, PHMG [19], and chloromethylisothiazolinone/methylisothiazolinone mixture [20,21].

Platelets play a critical role in thrombosis, the major complication of cardiovascular diseases such as myocardial infarction, angina pectoris, stroke, atherosclerosis, hypertension, and thromboembolism [22,23]. Platelets contribute to thrombosis by stimulating platelet aggregation [24,25] and coagulation cascade [26,27]. Platelets can trigger procoagulant activity by exposing phosphatidylserine (PS) to the extracellular side, providing sites for the assembly of clotting factors and activating them to induce thrombin generation and clot formation [28,29]. However, to our knowledge, little is known about the procoagulant activities of PHMG-p in platelets.

In the present study, we elucidated the effects of PHMG-p, the major component of HD, on procoagulant activity in platelets. PHMG-p induced PS exposure and thrombin generation, and the underlying mechanism was identified. We investigated the procoagulant effects of PHMG-p to provide a novel insight into HD-associated cardiovascular diseases.

## 2. Materials and Methods

### 2.1. Materials

PHMG-p was obtained from BOC Sciences (Shirley, NY, USA). Ethylenediaminetetraacetic acid (EDTA), Ethylene-bis(oxyethylenenitrilo)tetraacetic acid (EGTA), and catalase (CAT) were obtained from Sigma-Aldrich (St. Louis, MO, USA). Purified human factor Xa and factor Va were purchased from Prolytix (Essex Junction, VT, USA), and purified human prothrombin was obtained from Enzyme Research Laboratories (South Bend, IN, USA). Chromogenic substrate for thrombin was purchased form MyBioSource (San Diego, CA, USA) and purified human thrombin was obtained from Merck Millipore (Burlington, MA, USA). Phycoerythrin-labeled hamster anti-mouse CD61 (anti-CD61-PE) was purchased from BD Biosciences (Bergen County, NJ, USA), and fluorescein isothiocyanate-labeled annexin V (annexin V-FITC) was obtained from Biolegend (San Diego, CA, USA). 5-(6)-Chloromethyl-2′,7′-dichlorodihydrofluorescein diacetate, acetyl ester (CM-H_2_DCFDA), MitoSOX^TM^ Red, Fluo-4 acetoxymethyl ester (Fluo-4 AM), and BCA protein assay kit were purchased from Thermo Fisher Scientific (Rockford, IL, USA). Dihydroethidium (DHE), tetramethyl rhodamine ethyl ester (TMRE), and adenosine triphosphate (ATP) detection assay kit were obtained from Cayman Chemical (Ann Arbor, MI, USA). 1-Palmitoyl-2-[6-[(7-nitro-2-1,3-benzoxadiazole-4-yl)amino]caproyl]-sn-glycero-3-phosphatidylserine (C6-NBD-PS) and 1-oleoyl-2-[6-[(7-nitro-2-1,3-benzoxadiazole-4-yl)amino]hexanoyl]-sn-glycero-3-phosphatidylcholine (C6-NBD-PC) were obtained from Avanti Polar Lipids (Alabaster, AL, USA). All other reagents used were of the highest purity available.

### 2.2. Preparation of Platelets

All the protocols were approved by the Kangwon National University Animal Care and Use Committee (KW-230920-5). Male Sprague-Dawley rats (Envigo: Koatech, Pyeongtaek, Republic of Korea) weighing 250 to 300 g were used for the study. The animals were used after 1 week acclimation, and food and water were provided ad libitum. After anesthesia, blood was collected from the abdominal aorta with acid citrate dextrose (ACD, 85 mM trisodium citrate, 71 mM citric acid, 111 mM glucose, 1:6). Washed platelets were prepared by differential centrifugation. Whole blood was centrifuged at 250 g for 15 min, and platelet-rich plasma (PRP) was collected. PRP was mixed with Tyrode buffer (134 mM NaCl, 2.9 mM KCl, 1.0 mM MgCl_2_, 10 mM HEPES, 5 mM glucose, 12 mM NaHCO_3_, 0.34 mM Na_2_HPO_4_, pH 7.4) containing 1 μM prostaglandin E_1_ (PGE_1_), 0.2 U/mL apyrase, and 10% ACD and pelleted by centrifugation at 1000 g for 5 min. Platelet pellet was resuspended with Tyrode buffer containing 1 μM PGE_1_, 0.2 U/mL apyrase, and 10% ACD. After centrifugation at 1000× *g* for 5 min, platelets were resuspended in Tyrode buffer containing 0.3% bovine serum albumin (BSA) with a cell count of 1 × 10^8^ cells. CaCl_2_ was added to adjust 2 mM prior to use. 

### 2.3. Flow Cytometric Analysis

Measurements of PS exposure, reactive oxygen species (ROS) generation, mitochondrial membrane potential, intracellular calcium level, and caspase-3 activity were performed by flow cytometric analysis. Data from 10,000 events were collected and analyzed on a FACSVerse flow cytometer (BD Bioscience).

PS exposure was determined by using annexin V-FITC to detect PS and anti-CD61-PE to identify platelets. PHMG-p-exposed platelets were stained with anti-CD61-PE and annexin V-FITC for 30 min at room temperature in the dark and analyzed on the flow cytometer. Negative controls for annexin V binding were stained with annexin V-FITC in the presence of 2.5 mM EDTA, and platelets were considered to be positive when FITC fluorescence intensity was >99% of the signal from the EDTA negative control group.

ROS generation in platelets was detected with oxidant-sensing fluorescent probes, CM-H_2_DCF-DA, DHE, and MitoSOX^TM^ Red. Platelets were preloaded with 5 μM CM-H_2_DCF-DA, DHE, and MitoSOX^TM^ Red for 30 min at 37 °C in the dark and washed to exclude excessive probes. Preloaded platelets were treated with PHMG-p and analyzed on the flow cytometer.

Mitochondrial membrane potential was determined with the cell-permeant fluorescent dye TMRE. After PHMG-p exposure, platelets were incubated with 0.1 μM TMRE for 30 min at 37 °C and analyzed on the flow cytometer.

Fluorescent calcium indicator Fluo-4 AM was used to measure intracellular calcium level. Platelets were preloaded with 5 μM Fluo-4 AM for 30 min at 37 °C in the dark and washed to remove excessive Fluo-4 AM. Fluo-4-loaded platelets were incubated with PHMG-p and analyzed on the flow cytometer.

Caspase-3 activity was measured with capase-3/7 detection reagent, a fluorogenic substrate of activated casapase-3. After platelets were treated with PHMG-p, they were incubated with the caspase-3/7 detection reagent for 30 min at 37 °C in the dark. Platelets were analyzed on the flow cytometer.

### 2.4. Measurement of Intracellular ATP Level

Intracellular ATP level was determined using the ATP detection assay kit according to the provided procedure. Briefly, PHMG-p-treated platelets were centrifuged at 6000× *g* for 1 min at 4 °C. Platelet pellet was lysed with ATP detection sample buffer. ATP level was measured by luciferin/luciferase assay in Spectramax i3 (Molecular Devices, Sunnyvale, CA, USA) and normalized by protein content measured with BCA protein assay kit.

### 2.5. Detection of Phospholipid Translocation

The activity of phospholipid transporters was measured by translocation of C6-NBD-PC for scramblase activity and C6-NBD-PS for flippase activity. Platelets were treated with PHMG-p and then incubated with 0.5 μM C6-NBD-PC or C6-NBD-PS for 10 min at 37 °C. An aliquot from the platelets was diluted with Tyrode buffer with or without 1% BSA and stored in ice for 10 min. Samples were centrifuged at 5000× *g* for 1 min and lysed with 1% triton X-100. The fluorescence intensity (ex. 485 nm, em. 535 nm) of lysate was measured in Spectramax i3, and fluorescence intensity was compared before and after back extraction by BSA.

### 2.6. Thrombin Generation Assay

PHMG-p-treated platelets were incubated with factor Xa and 10 nM factor Va in HEPS-buffered saline (21 mM HEPES, 137 mM NaCl, 5 mM KCl, 0.7 mM Na_2_HPO_4_, 5.5 mM glucose, 2 mM CaCl_2_, 0.3% BSA, pH 7.2) for 5 min at 37 °C. Thrombin generation was initiated with 2 μM prothrombin for 5 min. An aliquot of the suspension was added to stop buffer (50 mM Tris-HCl, 120 mM NaCl, 2 mM EDTA, pH 7.9). Thrombin activity was determined using the chromogenic substrate for thrombin by measuring absorbance at 405 nm with a standard curve made with active-site-titrated thrombin.

### 2.7. Statistical Analysis

All experimental data were presented as the mean and standard error (SE). The data were subjected to Student’s t-test and one-way analysis of variance followed by Duncan’s post hoc test using SPSS 26 software. In all cases, *p* value of <0.05 was considered statistically significant.

## 3. Results

### 3.1. PHMG-p-Induced PS Exposure in Platelets

**Figure 1:**
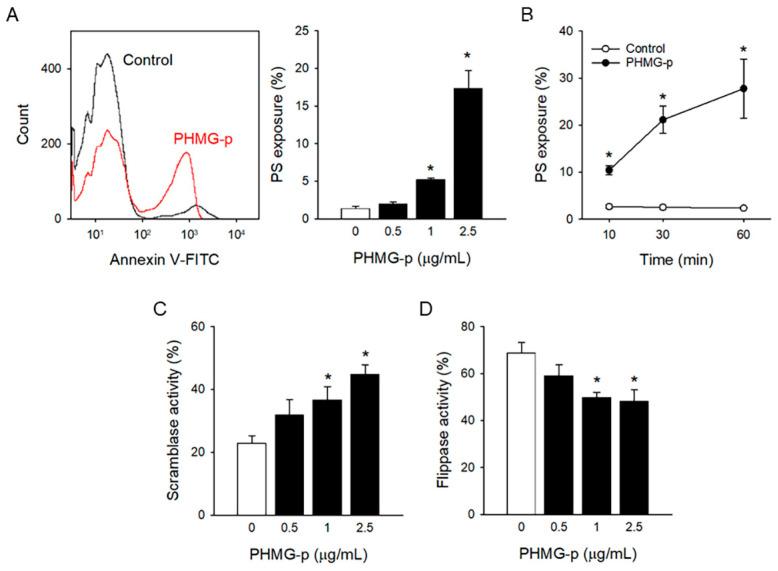
PHMG-p-induced PS exposure in platelets. (**A**) After platelets were incubated with PHMG-p for 1 h, PS exposure was measured. (**B**) Time-dependent PS exposure caused by PHMG-p (2.5 μg/mL) is shown. After treatment of PHMG-p for 1 h, (**C**) scramblase activity and (**D**) flippase activity were determined. Data are presented as mean ± SEM of 3–5 experiments. * *p* < 0.05 from control group.

To investigate the effects of PHMG-p on procoagulant activation of platelets, freshly isolated platelets were treated with PHMG-p for 1 h, and PS exposure, a contributing factor to blood clotting, was determined. PHMG-p induced PS exposure in a concentration- and time-dependent manner (Figure 1A,B). In addition, PS exposure increased even after 10 min of exposure of 2.5 μg/mL PHMG-p.

Phospholipid asymmetry in plasma membrane is precisely regulated by phospholipid transporters, scramblase, and flippase [30]. Scramblase disrupts phospholipid asymmetry through non-specific scrambling between lipid bilayers, which results in PS exposure. On the other hand, flippase maintains phospholipid asymmetry by flipping anionic phospholipids like PS to the inner leaflet, which results in restoring PS exposure. PHMG-p induced scramblase activity, measured by increased C6-NBD-PC translocation (Figure 1C), whereas it inhibited flippase activity, measured by reduced C6-NBD-PS translocation (Figure 1D) in a concentration-dependent manner.

### 3.2. Oxidative Stress Induced by PHMG-p

**Figure 2:**
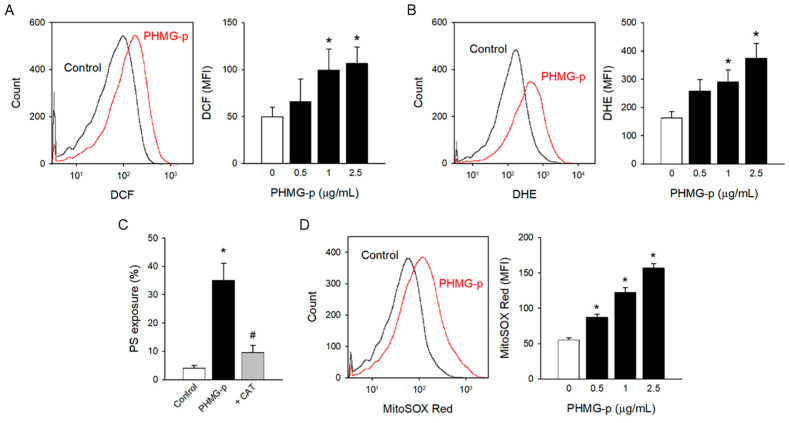
Oxidative stress induced by PHMG-p. (**A**) CM-H_2_DCF-DA-loaded platelet was incubated with PHMG-p for 1 h, and DCF fluorescence was detected on the flow cytometer. (**B**) DHE-loaded platelet was incubated with PHMG-p for 1 h and analyzed on the flow cytometer. (**C**) Platelets were incubated with CAT (1000 U/mL) for 30 min before PHMG-p (2.5 μg/mL) treatment, and PS exposure was determined. (**D**) MitoSOX-Red-loaded platelet was incubated with PHMG-p for 1 h, and MitoSOX Red fluorescence was detected on the flow cytometer. Data are presented as mean ± SEM of 4–6 experiments. * *p* < 0.05 from control group. # *p* < 0.05 from PHMG-p-treated group.

ROS generation has been suggested as a possible mechanism of PHMG-p-induced toxic responses [31,32,33,34]. To investigate the underlying mechanism of PS exposure in platelets, ROS generation was measured with oxidant-sensing fluorescent probes. PHMG-p enhanced ROS generation as measured by DCF fluorescence (Figure 2A). PHMG-p-induced ROS generation was confirmed with another common ROS probe, DHE (Figure 2B). Notably, PHMG-p-induced PS exposure was significantly attenuated by a pretreatment of antioxidant enzyme CAT, implying that ROS generation plays a pivotal role in PHMG-p-induced PS exposure (Figure 2C).

Mitochondria are regarded as the major source of ROS [35,36]. Furthermore, mitochondrial ROS has been suggested to play a critical role in platelet activation including PS exposure [37,38,39,40]. To investigate the effects of PHMG-p on mitochondrial ROS generation, MitoSOX Red mitochondrial superoxide indicator was used. Mitochondrial ROS generation significantly increased with PHMG-p (Figure 2D), implying that mitochondrial ROS may be involved in PS exposure caused by PHMG-p in platelets.

### 3.3. Intracellular Mechanism Underlying PHMG-p-Induced PS Exposure

**Figure 3:**
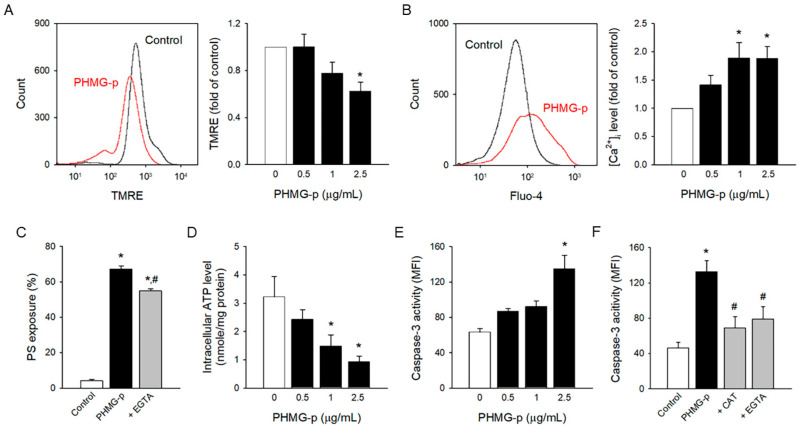
Intracellular mechanism underlying PHMG-p-induced PS exposure. (**A**) PHMG-p-exposed platelets were incubated with TMRE and analyzed in the flow cytometer. (**B**) Fluo-4-loaded platelets were treated with PHMG-p for 1 h, and Fluo-4 fluorescence was detected. (**C**) Platelets were incubated with EGTA (5 mM) for 30 min before PHMG-p (2.5 μg/mL) treatment, and PS exposure was determined. (**D**) Platelets were incubated with PHMG-p for 1 h, and intracellular ATP level was determined. (**E**) PHMG-p-exposed platelets were incubated with caspase-3/7 detection reagent and analyzed on the flow cytometer. (**F**) Platelets were incubated with CAT (1000 U/mL) or EGTA (5 mM) for 30 min before PHMG-p (2.5 μg/mL) treatment, and caspase-3 activity was determined. Data are presented as mean ± SEM of 3–5 experiments. * *p* < 0.05 from control group. # *p* < 0.05 from PHMG-p-treated group.

Oxidative stress can lead to the collapse of mitochondrial membrane potential, which is a critical step in mitochondrial dysfunction [41,42]. PHMG-p-altered mitochondrial membrane potential was measured by TMRE fluorescence (Figure 3A), an indicator of mitochondrial dysfunction.

Mitochondrial dysfunction may result in intracellular ATP depletion, intracellular calcium increase [43,44], and caspase-3 activation [45], which are known to be key events regulating PS exposure [30,46,47]. Intracellular calcium and caspase-3 stimulate scramblase activity and downregulate flippase, which results in PS exposure. Flippase is regulated in an ATP-dependent manner. PHMG-p significantly enhanced the intracellular calcium level in a concentration-dependent manner (Figure 3B), which matches well with the results of phospholipid transporters, scramblase, and flippase. PHMG-p-induced PS exposure was significantly attenuated by calcium chelator EGTA (Figure 3C), indicating the contribution of intracellular calcium. Consistently, PHMG-p depleted intracellular ATP (Figure 3D), which correlated well with the inhibition of flippase activity. Of note, PHMG-p promoted caspase-3 activation (Figure 3E), a typical apoptotic event, which may contribute to PHMG-p-induced PS exposure. Interestingly, CAT and EGTA reversed PHMG-p-induced caspase-3 activation (Figure 3F), indicating that caspase-3 activation was a secondary event mediated by ROS generation and intracellular calcium influx.

### 3.4. Procoagulant Effects of PHMG-p

**Figure 4:**
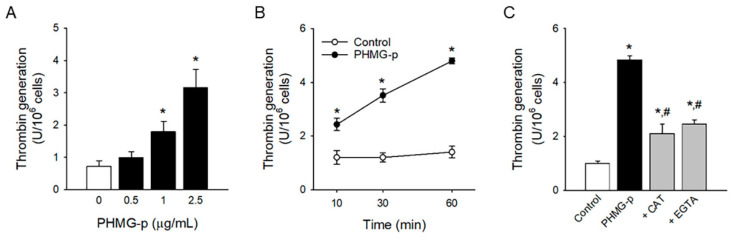
Procoagulant effects of PHMG-p. (**A**) After platelets were incubated with PHMG-p for 1 h, thrombin generation was measured. (**B**) Time-dependent thrombin generation caused by PHMG-p (2.5 μg/mL) is shown. (**C**) Platelets were incubated with CAT (1000 U/mL) or EGTA (5 mM) for 30 min before PHMG-p (2.5 μg/mL) treatment, and thrombin generation was determined. Data are presented as mean ± SEM of 3–5 experiments. * *p* < 0.05 from control group. # *p* < 0.05 from PHMG-p-treated group.

PS-exposing platelets can accelerate thrombin generation at the procoagulant surface and actively participate in the blood coagulation cascade. PHMG-p significantly enhanced thrombin generation in a concentration- and time-dependent manner (Figure 4A,B) in accordance with PHMG-p-induced PS exposure. Of note, the antioxidant enzyme CAT and calcium chelator EGTA reversed PHMG-p-induced thrombin generation (Figure 4C), confirming the role of ROS generation and intracellular calcium increase in the procoagulant effects of PHMG-p.

## 4. Discussion

In the present study, we demonstrated that PHMG-p, one of the major ingredients of HD, significantly increased PS exposure, resulting in enhanced procoagulant activity in platelets. PHMG-p induced ROS generation, leading to mitochondrial dysfunction, further intracellular calcium increase, intracellular ATP depletion, and caspase-3 activation. These intracellular events altered phospholipid transporters, scramblase, and flippase, which culminated in PS exposure. We believe that this study might provide an important clue for understanding HD-associated cardiovascular diseases. 

Although several follow-up studies of HD-exposed victims have suggested the possibility of a relationship between HD exposure and cardiovascular diseases [11,13,18], few studies have demonstrated the effects of the major components of HD on the cardiovascular system. Do et al. [20] and Kim et al. [21] reported that CMIT/MIT mixture causes functional damage in vascular smooth muscle and the endothelium. In another study with zebrafish, PHMG-p exposure enhanced fibrosis in bulbous artery [19]. However, these studies focused only on blood vessels, and little is known about the effects on platelets and prothrombotic risks, another counterpart in the development of cardiovascular diseases. Paliienko et al. [48] suggested that PHMG hydrochloride induced plasma membrane depolarization in platelets at the 5–100 ppm (5–100 μg/mL) level, a relatively high concentration, through the disruption of membrane lipids by interactions with Na^+^,K^+^-ATPase, which are non-specific events. The inhibition of membrane Na^+^,K^+^-ATPase may contribute to intracellular calcium mobilization and further platelet procoagulant responses [49]. However, the effects on membrane Na^+^,K^+^-ATPase were not fully clarified. We demonstrated that PHMG-p enhanced procoagulant activity mediated by platelets at a lower concentration (1 μg/mL). Moreover, considering the time-dependent effects of PHMG-p-induced PS exposure and thrombin generation (Figure 1B and Figure 4B), procoagulant effects may manifest at much lower levels with chronic and repetitive exposure. These results could suggest the possibility of prothrombotic risks after PHMG-p exposure.

The antimicrobial effects of PHMG are due to its interaction with membrane phospholipids, leading to membrane disruption [6,7,8]. Similarly, the disturbance of the plasma membrane and toxicity have been observed in several mammalian cells including lung epithelial cells, lung fibroblasts, monocytes, smooth muscle cells, platelets, and nerve tissue cells [48,50]. However, several studies have suggested apoptotic events represented as PS exposure for possible mechanism of toxicity in lung epithelial cells [33,51,52] and liver cells [53]. Consistently, PHMG-p enhanced PS exposure in platelets and resulted in triggering thrombin generation in our study. Of note, PHMG-p induced PS exposure at a similar concentration to that in previous studies. Moreover, PHMG-induced PS exposure was observed even after 10 min exposure (Figure 1B), whereas PS exposure was observed after 6- 36hr exposure in other studies.

It has been reported that there are two distinct pathways regulating platelet PS exposure, resulting in the procoagulant response and apoptosis [54,55]. A sustained high intracellular calcium level is suggested to be the key event to regulate PS exposure in procoagulant platelets [27,56], which correlates well with our results. However, it has also been reported that the apoptotic pathway may also participate in the procoagulant function through caspase-3 activation [57,58,59]. Schoenwaelder et al. reported that the proapoptotic agent ABT-737 enhanced procoagulant activity in platelets through caspase-3 activation [57]. In addition, the apoptotic pathway including caspase-3 activation was involved in the procoagulant response by chemicals, namely, anti-cancer agent doxorubicin and phytochemical dipsacus saponin C [58,59]. Of note, PHMG-p triggered caspase-3 activation, which may also be involved in the procoagulant responses by PHMG-p.

Several previous studies have suggested that PHMG may prompt cell death by directly triggering apoptosis [33,51,52,53]. Jung et al. [51] demonstrated that PHMG hydrochloride induced apoptosis by altering gene expression. Park et al. [52] suggested membrane and organelle damage as the key initiator of PHMG-p-induced apoptosis. Park et al. [33] demonstrated that PHMG-p-induced DNA damage caused apoptosis. Kim et al. [53] reported that PHMG-p enhanced apoptosis via induction of endoplasmic reticulum stress. Notably, the typical apoptotic event, caspase-3 activation, was abolished by antioxidant enzyme CAT and calcium chelator EGTA in the present study. PHMG-p-induced apoptotic events could be a secondary event mediated by ROS generation and intracellular calcium elevation, rather than PHMG-p directly activating apoptotic pathways. Similarly, the dipsacus-saponin-C-induced apoptotic events, Bax/Bak translocation and caspase-3 activation, were reversed by calcium chelator EGTA.

To investigate the toxicity of PHMG-p through HD exposure, the blood level of PHMG-p in HD-exposed groups or animals need to be compared. Unfortunately, the measurement of PHMG-p level in living organisms is difficult as PHMG-p is a mixture of polymers with distinct molecular weights. This limits the use of common analytic detection methods such as mass spectroscopy, UV, and fluorescence detection. To overcome this limitation, Mushtaq et al. [60] determined the biodistribution of PHMG in rats using radiolabeled PHMG. PHMG was primarily distributed in the lung after intratracheal instillation, but some translocated to other organs such as the liver, spleen, stomach, intestine, and kidney. This reveals that PHMG passed through the circulatory system, and that blood cells can be a potential target of inhaled PHMG.

One limitation of this study is that rat platelets were adopted instead of human platelets. Extensive study has been carried out with murine platelets to investigate the biology of human platelets due to the similarity between rat and human platelets [61,62]. However, rat platelets showed a distinct response to human platelets in a number of cases, raising concerns about using rat platelets instead of human platelets [63,64]. Therefore, the procoagulant effects of PHMG-p on platelets should be confirmed with human platelets in further research. The other limitation of this study is that the release of platelet-derived microparticles (MPs), another counterpart of procoagulant effects [65,66], was not studied. In addition to PS exposure, the shedding of PS-bearing MPs could also accelerate thrombin generation at the procoagulant surface and actively participate in thrombosis [67]. In future research, it would be meaningful to study the effects of PHMG-p on platelet-derived MP release and its physiological role.

In the present study, oxidative stress was identified to be the key event for PHMG-p-induced PS exposure and procoagulant activity in platelets. Many studies have demonstrated that PHMG can generate ROS in various systems, including in vitro cell lines, co-culture systems, and in vivo animal models. PHMG enhanced ROS generation in human lung epithelial A549 cells [33,34] and murine macrophage RAW264.7 cells, which resulted in decreased cell viability [32]. Kim et al. [31] observed ROS generation in an air–liquid co-culture model with human bronchial epithelial Calu-3 cells and monocyte THP-1 cells. PHMG-p-induced oxidative stress was also determined in in vivo animal models, namely, zebrafish in water containing PHMG [19] and rat lungs by PHMG inhalation [68]. Consistent with previous reports, PHMG-p enhanced cytoplasmic ROS and mitochondrial ROS generation in platelets. Importantly, antioxidant enzyme CAT abolished PHMG-p-induced PS exposure and thrombin generation, clearly confirming the role of oxidative stress in procoagulant activation by PHMG-p.

## 5. Conclusions

In conclusion, we demonstrated that PHMG-p can enhance procoagulant activity through PS exposure mediated by ROS generation, mitochondrial dysfunction, intracellular calcium increase, intracellular ATP depletion, and caspase-3 activation, altering phospholipid transporters. These events could contribute to prothrombotic risks, providing important information for understanding cardiovascular disease associated with HD exposure.

## Data Availability

The data presented in this study are available on request from the corresponding author.
